# Using a smartphone app to monitor Raynaud’s attacks and quantify skin colour changes—towards objective outcome measures for Raynaud’s

**DOI:** 10.1093/rheumatology/keaf141

**Published:** 2025-03-12

**Authors:** Graham Dinsdale, Andrea Murray, Joanne Manning, Mark Dickinson, Ariane L Herrick, Chris J Taylor

**Affiliations:** Rheumatology Department, Salford Royal Hospital, Northern Care Alliance NHS Foundation Trust, Salford, UK; Division of Musculoskeletal and Dermatological Sciences, The University of Manchester, Manchester Academic Health Science Centre, Manchester, UK; Rheumatology Department, Salford Royal Hospital, Northern Care Alliance NHS Foundation Trust, Salford, UK; Photon Science Institute, The University of Manchester, Manchester, UK; Rheumatology Department, Salford Royal Hospital, Northern Care Alliance NHS Foundation Trust, Salford, UK; Division of Musculoskeletal and Dermatological Sciences, The University of Manchester, Manchester Academic Health Science Centre, Manchester, UK; Centre for Imaging Sciences, Institute of Population Health, The University of Manchester, Manchester, UK

**Keywords:** outcome measure, smartphone photography, smartphone app, Raynaud’s phenomenon, systemic sclerosis

## Abstract

**Objectives:**

Our overall aim was to develop a smartphone app to collect photographic images of Raynaud’s phenomenon (RP) attacks alongside patient reported outcome measures (PROMs). Specific objectives included assessing the feasibility of patients documenting RP attacks using mobile phones, developing image analysis methods to document colour change, and comparing photographic parameters with ‘non-imaging’ app and paper diary parameters.

**Methods:**

In study 1, 36 patients with systemic sclerosis (SSc)-related RP photographed RP attacks over 15 days as well as completing an RP paper diary. In study 2, 40 patients with either SSc-related or primary RP used a smartphone app to collect, over 15 days, photographic images and data including frequency and severity of attacks. In both studies, mean colour change during each attack (indicating severity) was quantified by the Bhattacharyya distance.

**Results:**

In study 1, 24/36 (67%) patients completed the study of whom 22 photographed at least one RP attack (median number of attacks 12.5, range 1–53); 18/24 (75%) patients preferred phone to paper diary documentation. ‘Photographic’ and ‘paper diary’ frequency (but not severity) of attacks correlated strongly, with a correlation coefficient of 0.71 (95% CI: 0.41, 0.87); *P* = 0.002. In study 2, 36/40 (90%) completed the study, providing 1747 usable images from 456 RP attacks. ANOVA demonstrated that RP colour change was significantly different with different values of RP attack severity (*P* <0.001).

**Conclusion:**

Collecting photographs of RP attacks and PROMS via a smartphone app was feasible and preferred by patients to data collection via paper diary, providing proof-of-concept for validation studies of app-based outcome measures for RP.

Rheumatology key messages

- Taking hand photographs during RP attacks via a smartphone app is feasible for most patients.
- Magnitude of colour change assessed from photographs is associated with app-reported RP severity.
- Mobile phone technology has the potential of providing objective outcome measures for RP.

## Introduction

Raynaud’s phenomenon (RP) is an episodic, exaggerated vasospastic response to cold environments and/or emotional stress. Classically, patients experience triphasic colour change of the fingers and sometimes the toes: white/pallor, blue/cyanosis and red/hyperaemia [1]. RP is common, affecting around 5% of the general population [2], and is usually idiopathic (‘primary’ RP, PRP). RP falls within the province of the rheumatologist because RP can be secondary to connective tissue disease and is particularly severe in patients with systemic sclerosis (SSc), almost all of whom have RP [3]. RP has a major impact on quality of life not only in patients with SSc [4] but also often in those with PRP [5].

Current treatments for RP are often ineffective or only poorly effective [6–9]. New treatments are badly needed, yet clinical trials have been hampered by the lack of reliable outcome measures of frequency, duration and severity of RP attacks for use ‘in the field’ (including out-of-doors), which is where attacks commonly occur. The Raynaud’s Condition Score (RCS) [10, 11], where patients report their symptoms on a 0–10 Likert scale, assesses impact of frequency, duration and severity of RP attacks. Patient reported outcome measures (PROMs) are highly subjective, and subject to a marked placebo effect [12–14].

Smartphone technology could provide a way forward. Over the past 10 years, as smartphones with cameras have become commonplace, patients have started to bring photographs of their RP symptoms on their smartphones to show their doctor at clinic visits. A systematic method of capturing and interpreting such data would improve their value significantly and could provide a long-awaited objective outcome measure for use in phase II and phase III trials. To explore this we have conducted two connected studies. The first study (study 1) had the primary aim of assessing the feasibility of patients using mobile phone cameras to document RP episodes in everyday life. Secondary objectives included: (i) developing image analysis methods to quantify colour change in photographs of RP, and (ii) comparing image-derived data (number of RP attacks, ‘severity’ of colour change) with that collected from paper-based patient self-reports (RP paper diary). The second study (study 2) built on findings from the first (taking patient feedback into account): we developed a custom-developed smartphone app to assist patients with image (and other data) collection. The aim of study 2 was to compare the digital photograph parameters with the ‘non-imaging’ parameters collected on the app. Results would allow us to understand (for example), the degree of colour change that is clinically meaningful.

## Methods

### Study 1 (concept feasibility)

#### Patients

Thirty-six patients with RP secondary to SSc [15] (details in Table 1) were recruited at Salford Royal Hospital, a tertiary referral centre for patients with SSc. All participants gave informed, written consent. Study 1 was approved by the East Midlands—Nottingham 2 Research Ethics committee (reference: 14/EM/1043).

**Table 1.** Demographics and clinical characteristics of the patients included in study 1 and study 2

	Study 1: concept feasibility (*n* = 36)	Study 2: mobile app study (*n* = 40)
Primary RP, *n* (%)	N/A	8 (20)
Fulfilling ACR/EULAR 2013 criteria for SSc, *n* (%)	36 (100)	32 (80)
Female, *n* (%)	32 (89)	40 (100)
Age, median (range), years	55 (24–82)	57 (25–74)
Light skin tone, *n* (%)	34 (94)	39 (98)
Duration of RP, median (range), years	14 (2–37)	17 (0–53)
Disease duration since first non-RP clinical manifestation, median (range), years^a^	5 (1–35)	6 (0–39)
Previous intravenous vasodilator use, *n* (%)^a^	11 (31)	7 (18)
Current vasodilator use, *n* (%)		
CCB	20 (56)	12 (30)
ARB	4 (11)	1 (3)
ERA	1 (3)	1 (3)
ACEI	1 (3)	0 (0)
PDE5I	6 (17)	5 (13)
History of digital ulceration, *n* (%)^a^	17 (47)	12 (30)
Limited cutaneous SSc, *n* (%)^a^	27 (75)	29 (91)
Anti-centromere antibody positive, *n* (%)^a^	19 (53)	20 (63)
Anti-topoisomerase I antibody positive, *n* (%)^a^	6 (17)	6 (19)
Anti-RNA polymerase III antibody positive, *n* (%)^a^	3 (8)	0 (0)
Patient-estimated number of RP episodes per day at baseline, median (IQR)	2.5 (2.3)	2 (2.0)
Raynaud’s Condition Score at baseline, mean (s.d.)	4.0 (2.6)	3.7 (3.0)

ACEI: angiotensin-converting-enzyme inhibitor; ARB: angiotensin receptor blocker; CCB: calcium channel blocker; ERA: endothelin receptor antagonist; IQR: interquartile range; N/A: Not applicable; PDE5I: phosphodiesterase type 5 inhibitor; RP: Raynaud’s phenomenon; SSc: systemic sclerosis.

a Applies only to patients with SSc.

#### Study pathway

Patients attended twice. At visit 1 (day 0) the patient was asked to complete a questionnaire assessing their familiarity with various aspects of smartphone technology, followed by an RCS and an assessment of the number of their daily attacks. Patients were then trained by a member of the research team (G.D.) to photograph their hands (using their own mobile phone camera) during RP episodes (see Supplementary Table S1, available at *Rheumatology* online, for explicit photography instructions given to patients) and given an X-rite Colour Checker card (to assess necessity of colour correction under different lighting conditions) and a 14-day RP paper diary (to be completed daily, and including space to record number of attacks as well as the RCS). At visit 2 (day 15 or later) patients returned their completed RP diary and the X-rite Colour Checker card. Study photographs were downloaded from the patient’s smartphone to a laptop. Each patient also completed a questionnaire detailing their experiences during the study. This questionnaire included various items (1–10 scale) on ease of using the mobile phone to photograph RP attacks, as well as similar questions about using the RP paper diary. General questions asked the participants to state, separately, whether they would be willing to use the phone and the RP paper diary in future studies, and which, if either, they preferred.

#### Data organization and cleaning

Images were pseudonymized, catalogued by subject, reviewed to remove ‘poor quality’ images (those out of focus, or not including whole hands), and sub-sorted by aspect (dorsal/palmar) and episode (using image time stamp data).

#### Assessing feasibility

Feasibility of patients photographing their own RP episodes was assessed against three criteria: (i) successful ‘task completion’, i.e. the patient was able to record image(s) of their hands during an RP episode; (ii) how the photographic record (number of images/episodes recorded) compared with the RP paper diary record of number of attacks; and (iii) data from the patient preference questionnaire.

#### Image analysis

**Figure 1.**
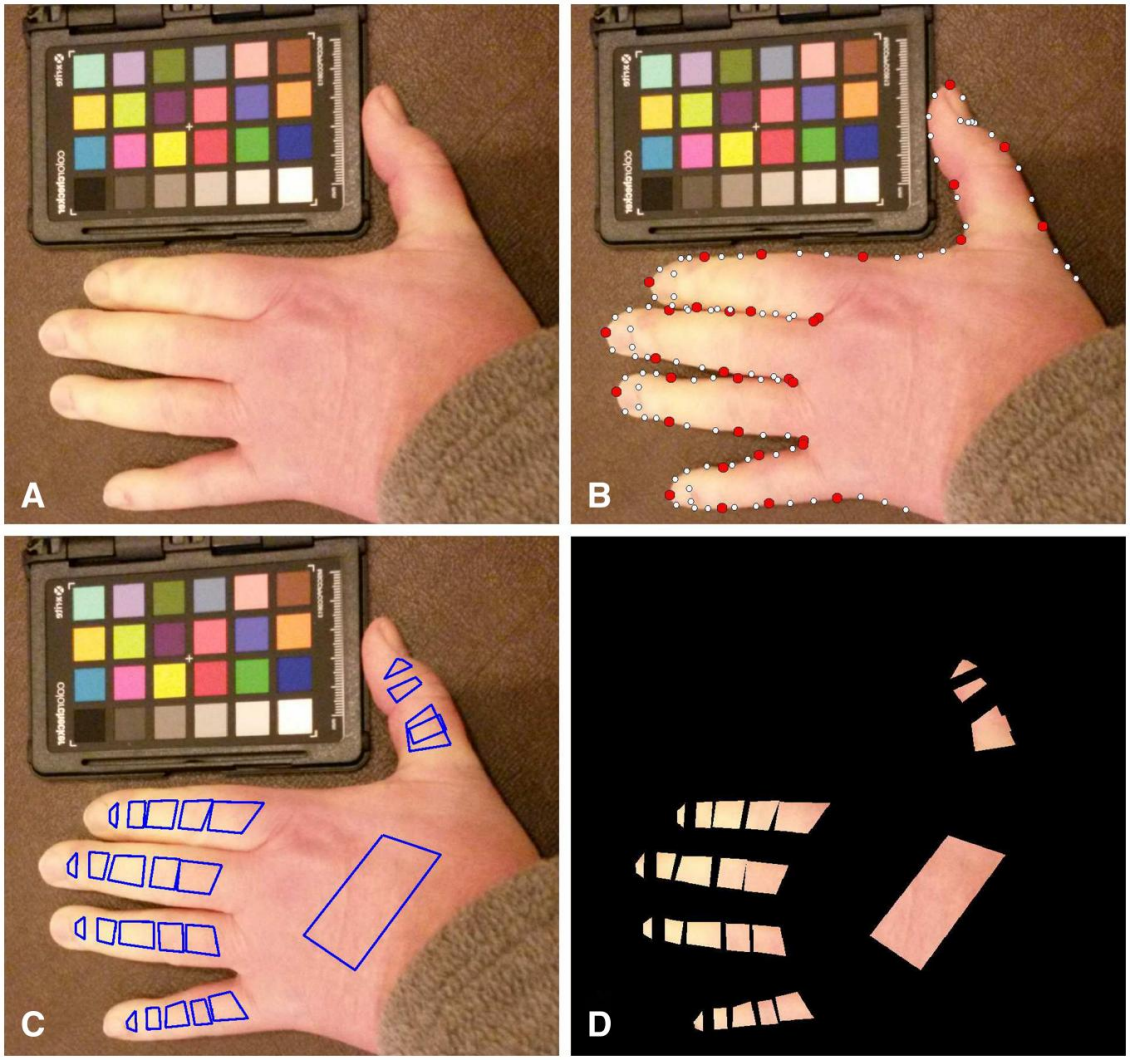
Hand mark-up and segmentation process, going from initial photograph to segmented digits and 25 defined regions of interest. (**A**) Original image; (**B**) hand border defined post-model fitting (red points are anatomical locations); (**C**) ROIs defined relative to points on model shown in (B); (**D**) masked original image showing just the ROIs defined for pixel extraction. The pixel values extracted from (**D**) are saved and processed as described in Fig. 2. ROI: region of interest

**Figure 2.**
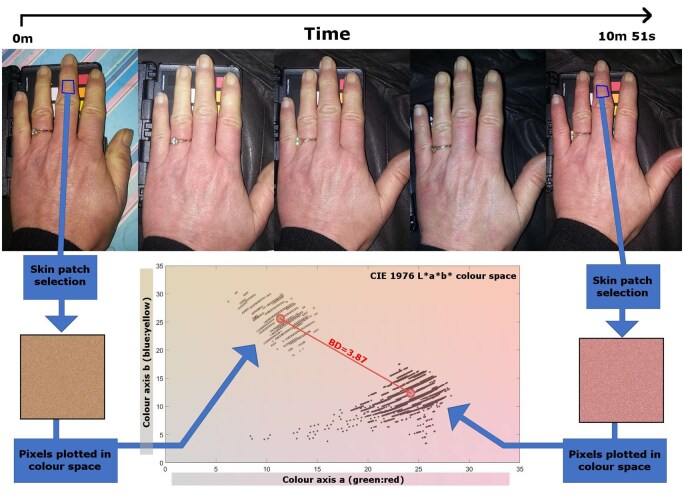
A visual demonstration of the connection between changes in skin colour change and changes in Bhattacharyya distance (BD), using one ROI as an exemplar. A sequence of hand images (top) throughout a Raynaud’s attack. The same region of interest (from identical anatomical locations) is extracted from the first image (coloured square, left) and the last image (coloured square, right). The colour values of all pixels within the ROI can be converted to L*a*b* colour space, with the hue components (red-green, blue-yellow, a and b) plotted for each ROI as shown in the graph (bottom). For the studies described here the BDs from all ROIs on the digits (calculated *vs* the dorsal ROI) were combined (averaged) in each image for time series analysis. CIE: Commission Internationale de l'Eclairage

The analysis of the images is described in detail in the Supplementary Data S1, available at *Rheumatology* online. The images were analysed ‘as taken’ by the smartphone camera (JPEG format) with no additional pre-processing. The steps taken were the following:

- The outline of the hand (Fig. 1A) was found automatically using BoneFinder software [16] (Fig. 1B).
- Twenty-five regions of interest (ROIs) were defined on the hand, 24 on the digits and one reference ROI on the dorsum (see Fig. 1C). The dorsum was chosen as a normal skin colour reference since it is as an area typically unaffected by RP attack processes. Variations in skin colour caused by RP were compared against the patient’s unaffected skin at the time of the episode, therefore controlling for other variables that may affect skin colour over longer periods (e.g. tanning).
- The ROIs were used to mask and segment the image (Fig. 1D). The red, green and blue (RGB) pixel colour values for all pixels in each ROI segment were then extracted automatically.
- The RGB pixel values were transformed into an alternative Commission Internationale de l'Eclairage (CIE) L*a*b* colour space [17] to facilitate further analysis. Figure 2 shows the distribution of pixel colour values for a typical ROI, plotted in the a* (green-red) *vs* b* (blue-yellow) dimensions of the colour space at two time points during an RP episode.
- The shift, or difference, in distributions of pixel colour values between each digit ROI and the reference ROI, was calculated using the Bhattacharyya distance (BD) [18, 19]. The BD is a statistical measure of similarity between two distributions of data (in this case colour values in CIE L*a*b* space). A BD of zero between two distributions suggests perfect overlap/similarity, with increasing dissimilarity as BD values increase towards infinity.

In analysing study 1 we looked at the colour change behaviour of groups of ROIs (specifically grouping all ROIs on all eight digits together) within episodes (made up of longitudinal image sequences) to assess whether the BD provides a useful measure of RP.

The image analysis methods developed for study 1 were also used for study 2.

#### Comparing image-derived data to paper diary data

Data on the number of attacks *imaged* (from image file time stamps) were compared with the patient-reported number of attacks from the RP paper diary. In addition (to compare attack severity) the mean BD per attack was compared with the RCS for that day from the RP paper diary. Pearson’s correlation was used to determine the relationship between the image-derived and paper-based metrics.

### Study 2 (mobile app study)

#### Patients

Forty patients either with PRP [20] or with RP secondary to SSc (Table 1) were recruited at Salford Royal Hospital. All participants gave informed, written consent. Study 2 was approved by the South Central NRES Committee Oxford A Research Ethics committee (reference: 17/SC/0671).

#### Study pathway

**Figure 3.**
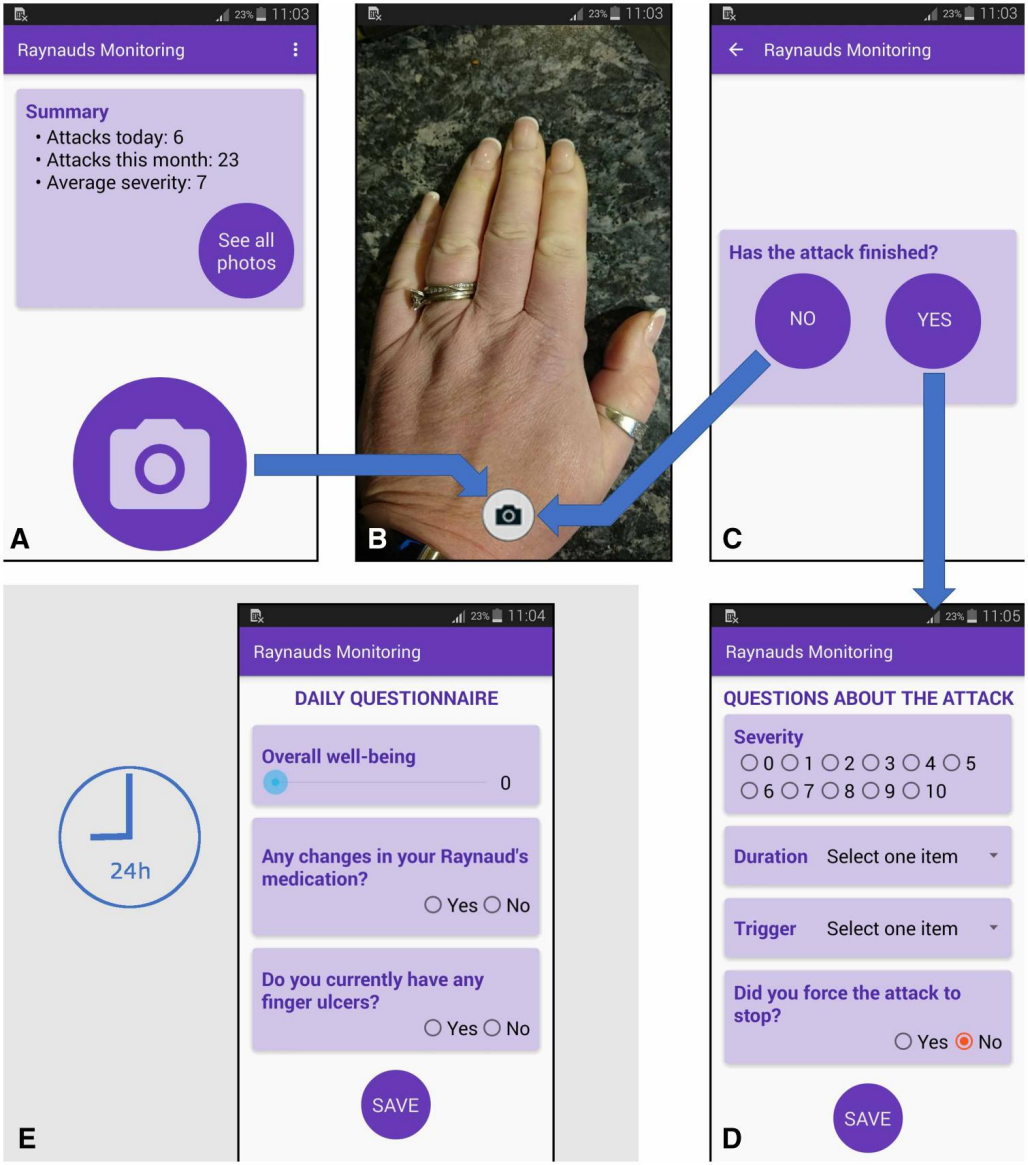
Screenshots from the Raynaud’s app interface. The main screen is shown in (**A**), with summary data about attacks, along with the possibility to review earlier images. To start imaging a new attack, the patient taps the large camera button. The app then redirects to the device’s camera app (**B**), where the patient frames and captures their hand image as instructed. The screen in (**C**) is then shown, asking the patient if the attack is finished. Answering ‘No’ sets a 1 min reminder on the device to take another image, and the process repeats *ad infinitum*. Answering ‘Yes’ on screen (**C**) ends the current attack and displays the attack questionnaire shown in (**D**). The patient completes the process by answering the questions and pressing ‘Save’. Additionally, the patient is asked to complete the daily questionnaire (**E**) once every 24 h (at a pre-set time of the patient’s choosing). See Supplementary Table S2, available at *Rheumatology* online, for full contents of post-attack questionnaire (**D**), and daily questionnaire (**E**)

The study pathway was similar to that for study 1, but instead of patients being asked to use their own mobile phone, they were given at their first visit an Android smartphone onto which a custom-developed mobile app had been pre-loaded (see Fig. 3, and ‘Development of a custom-developed smartphone app’ section below). On the app patients were asked to complete ‘post-attack’ and daily questionnaires (described in Supplementary Table S2, available at *Rheumatology* online). At the second study visit (day 15 or later) the patients returned the mobile device containing images and app data. Instructions for photographing RP episodes were as for study 1, except the X-rite Colour Checker cards were not used (see ‘Results’ section for explanation). The app enabled the patient to image their hands during RP episodes, and collect ‘non-imaging’ data including a per episode RP attack severity scale. All app data were downloaded from the device following visit 2. Participants also completed a questionnaire asking about their experience of using the app.

#### Development of a custom-developed smartphone app

A simple smartphone app was developed using the Android Studio developer suite of tools (available at http://developer.android.com) and side-loaded (by connecting directly to a PC rather than via an app store) onto handsets for use by study participants. App development was aided by feedback from a group of 10 patients, who were consulted several times. The patient group asked for a simple interface with large, conveniently positioned buttons. They also provided information/advice relating to some questionnaire options (e.g. attack triggers), agreed the attack imaging time interval, gave feedback on the questionnaire interface (e.g. buttons *vs* sliders), and agreed the app colour scheme. See Fig. 3 for more details.

In this study the app stored all data on the handset—at no point was data transferred wirelessly (via WiFi or cellular networks). Imaging data were stored in a separate folder on the device, with date-stamped subfolders for each day. Questionnaire data were stored in text files alongside the imaging data.

#### Comparison of digital image (photographic) parameters with ‘non-imaging’ app parameters

Images were analysed as for study 1, with ROIs identified and extracted automatically. The BD for each ROI was then calculated. The mean BD per attack (BD of all ROIs on the digits averaged across each image and for all images in an episode) was compared with the per attack severity score using *n*-way ANOVA. The within-app RP severity score is (like the RCS) a subjective measure of RP, with some patients always scoring highly (towards 10), with others minimizing RP attack severity (towards 0). An *n*-way ANOVA allows for testing of multiple factors on an outcome, in this case the contribution of patient perception of RP severity, as well as the RP severity score itself, to the mean BD. In order to assess the sensitivity of BD, it is further useful to know whether different severity score groups are associated with significantly different values of BD—this can be assessed using multiple comparison methods while applying appropriate compensation for performing multiple statistical tests (MATLAB’s ‘multcompare’ function with Tukey’s honestly significant difference criterion).

Correlations of raw BD data with severity score were done using Pearson’s correlation, as in study 1.

## Results

### Study 1: feasibility

All patients were familiar with smartphones and had used them to take photographs and browse the internet, with just one patient not owning a smartphone-type mobile device at the time of the study.

#### Task completion

Of the 36 patients recruited into the study, 24 (67%) completed the study and returned one or more images of their hands. Of the 12 non-completers, 10 were lost to follow-up due to being unable or unwilling to return to the hospital for the required second study visit within the overall time frame of the project, and two were unable to complete due to worsening health not connected with the study.

For the 24 patients completing the study, 22/24 (92%) were able to photograph at least one RP episode (defined as two or more images within a sliding 30 min window), with a median (range) of 12.5 (1–53) episodes photographed per patient. The 24 patients returned a median (range) of 30 (7–270) images each, 1205 images in total. Following assessment of images for usability (good focus, relevance, etc.), 792/1205 images were suitable for further analysis.

#### Comparison of photographic report to RP paper diary

This is described below.

#### Patient preference questionnaire

On a scale from 1 (Very Easy) to 10 (Very Difficult), 20/24 patients (83%) gave answers between 1 and 3 in response to the query ‘Overall experience of using the mobile phone to document your Raynaud’s attacks’. Of the four patients giving higher (i.e. more negative) answers, three left additional text comments mentioning specific difficulties with their own hand function. When asked to express a preference for using their mobile phone or paper diary, 18 (75%) preferred their phone, 2 (8%) the paper diary and 4 (17%) expressed no preference. When participants were asked if they would be willing to use their phone to record information about their Raynaud’s in future 21 (88%) said ‘Yes’, while only 15 (63%) said the same for the paper diary.

### Study 1: image analysis

The 792 images that were assessed suitable for further analysis were further divided into palmar (148 images) and dorsal (644 images) aspect views. It was decided to concentrate on only dorsal views for this and subsequent analysis, mainly due to the more consistent presentation of the images. An analysis of colour correction using the X-Rite Colour Checker cards was performed on all images (792 total) where the card was visible. Colours extracted from corrected images were compared with those from the uncorrected original image. This demonstrated that, under the imaging conditions used by the study participants, the Colour Checker card colour correction was unnecessary. Any colour adjustments were small compared with the colour changes involved in RP episodes (see Supplementary Fig. S1, available at *Rheumatology* online).

### Study 1: comparing image-derived data with paper diary data

#### Number of RP attacks

**Figure 4.**
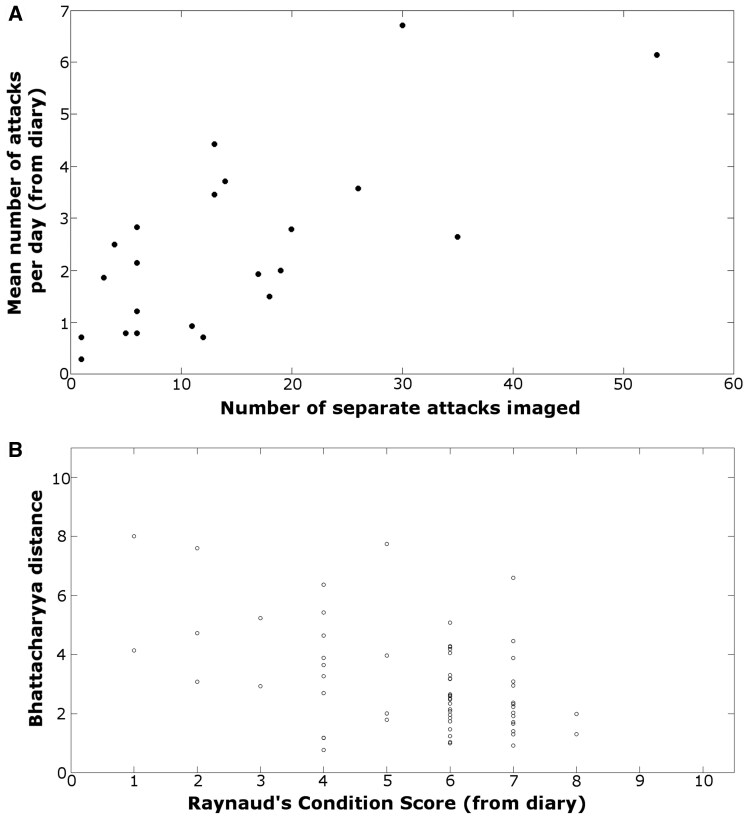
Results from study 1. (**A**) Number of episodes recorded in paper diary *vs* number of episodes imaged. (**B**) Mean BD per attack *vs* daily RCS from RP paper diary. BD: Bhattacharyya distance; RCS: Raynaud’s Condition Score; RP: Raynaud’s phenomenon

The 24 patients recorded in the RP paper diaries a median (range) of 2 (0–12) episodes per day over the 14-day study period. The number of RP episodes imaged, and those recorded in the RP paper diary were strongly correlated: *r* = 0.71 (95% CI: 0.41, 0.87); *P* = 0.002 (Fig. 4A).

#### RP attack severity

As described previously, the within-image BD was calculated for each ROI. The median (range) paper diary RCS over the study period was 2 (0–9). The relationship between mean BD values per episode (averaged over all ROIs and all images within an attack sequence) with RCS is shown in Fig. 4B: these were negatively correlated (correlation coefficient −0.48 [95% CI: −0.7, 0.18]; *P* = 0.003). A larger BD indicates a larger change in skin colour over time or between sites depending upon the comparison; thus, a negative correlation signifies that as colour changes increase the RCS decreases. It should be noted that the RCS was recorded by participants every day during the study, regardless of whether they had any RP episodes or, if they did, whether they photographed them or not. This results in low median values—the RCS values represented in Fig. 4B relate only to those days when attacks were imaged.

### Study 2

Of the 40 patients recruited into study 2, 36 (90%) completed imaging and both study visits. Of the four participants who did not complete the study, two (5%) reported no RP episodes during the study period and two (5%) did not complete the study tasks for personal reasons.

#### Patient questionnaire

On a scale from 1 (Very Easy) to 10 (Very Difficult), 33 patients (92%) gave answers between 1 and 4 in response to the query ‘Overall experience of using the mobile phone to document your Raynaud’s attacks’.

### Study 2: comparing the image-derived data to non-imaging app data

#### Number of RP attacks

For the 36 patients completing the study, there were a total of 751 episodes recorded on the app with one or more image recorded. The median (range) number of episodes per patient was 11 (1–67). A total of 2988 images were taken during episodes, with a median (range) of 2 (1–41) images per episode. From these a total of 1747 usable images from 456 episodes were taken forward for further analysis.

#### Comparing image severity (as assessed by BD) to RP attack severity (as assessed via app)

**Figure 5.**
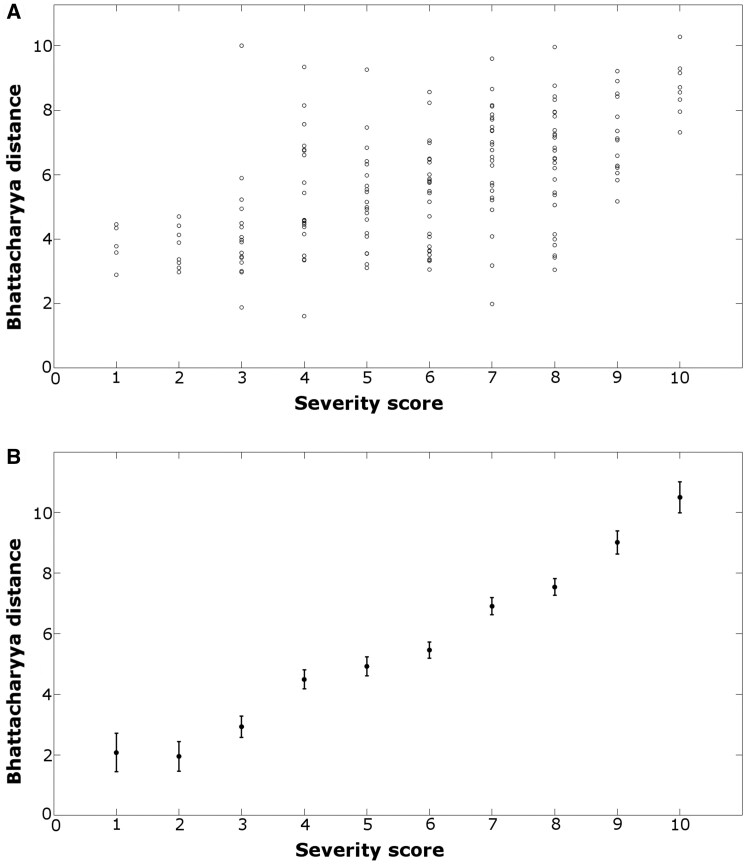
Comparisons of BD and RP per attack severity. (**A**) Raw data for mean BD *vs* RP per attack severity score. (**B**) Following ANOVA (which effectively takes into account the variations in usages of the RP severity score), the relationship between RP per attack severity *vs* BD. The intervals shown around each estimate are computed such that, when comparing two values of RP severity, the estimates can be considered significantly different if the intervals do not overlap. BD: Bhattacharyya distance; RP: Raynaud’s phenomenon

The median RP severity reported via the app was 6 (inter-quartile range: 4), while the mean value of the BD was 5.4 (s.d. 4.1). The raw data for the BD *vs* RP per attack severity score (Fig. 5A) showed minimal correlation (correlation coefficient −0.21). However, using ANOVA showed that measured values of the mean image BD were significantly different when different values of RP severity were recorded by the patient (*P* <0.001), i.e. attacks where patients selected different values of RP severity had significantly different values of BD. ANOVA effectively corrects for the individual patients’ subjective use of the severity scale and shows that when choosing a higher severity score for one attack *vs* another, this is associated with an increase in the measured BD. Fig. 5B shows the relationship between BD and the severity score after ANOVA. The error bars on the data points show the uncertainty in the BD associated with a specific severity score, but also allow comparison between severity scores. Where there is overlap between the error bars on two severity scores (e.g. score 1 *vs* score 2) then the BD values are not significantly different and therefore the colour changes are similar. However, when there is no overlap between the error bars (e.g. score 6 *vs* score 7), then the BD values are significantly different, and the colour changes will be distinguishable. Across all attacks/patients the *F*-value from ANOVA for RP severity was 76.2, suggesting that the variation in BD for different values of RP severity is much greater than the variation in BD for any one value of RP severity.

## Discussion

The key findings from this study are that collecting mobile phone images during RP attacks was feasible for the majority of patients (many of whom had severe SSc-related finger problems), that patients preferred documenting Raynaud’s attacks via the smartphone app to using a paper diary, and that the magnitude of ‘photographic’ colour change differed between different RP app-reported attack severities. Together these findings suggest that smartphone app imaging has strong potential as an outcome measure for RP.

We are not aware of any previous studies in which patients have photographed their RP-associated colour changes. RP is a complex construct, but is defined by colour change, and so some objective measure of that colour change would seem ideal as a much-needed outcome measure to facilitate clinical trials. Attacks of RP occur when patients are ‘out and about’ and so measures of the colour change of RP need to be collected throughout the day, when attacks occur. Up until recently, this has not been possible, but smartphones now provide this opportunity. That 88% of patients stated that they would be willing to use their phone to record information about their RP in future studies is a persuasive argument to progress towards validation as an outcome measure/set of outcome measures. Individuals almost always have their mobile phone to hand, unlike a paper diary, which is often ‘left behind’ or lost, leaving the patient to rely on their recall of attacks at some later time point as opposed to immediate documentation on the app.

‘Photographic severity’ as measured by the BD did not correlate with the daily RCS (study 1) but did differentiate between different app-reported attack severities, suggesting that there may be advantages in considering the severity of RP attacks separately rather than averaging over a 24-h period.

It is well recognized that current measures of outcome in studies of RP are inadequate. OMERACT (Outcome Measures in Rheumatology) recognizes the importance of developing such outcome measures for SSc-related RP [21]. The 27-item Assessment of Systemic Sclerosis-Associated Raynaud's Phenomenon (ASRAP) questionnaire, and a 10-item short form [22, 23] are novel PROMS that have been developed with extensive patient input, have excellent repeatability, and capture experiences not assessed by ‘traditional’ RP diaries [24]. However, all PROMS have the inherent problem of subjectivity. For early phase proof-of-concept studies there is increasing interest in laboratory-based non-invasive imaging methods such as laser Doppler imaging/laser speckle contrast imaging and thermography, usually incorporating response to a cold challenge [25–27], but these are not going to be feasible for later phase community-based, multicentre randomized controlled trials. Our smartphone app allows incorporation of an objective measure (colour change) alongside PROMs and other data relevant to RP attacks.

Our study had limitations. Patient numbers were relatively small but did include patients with severe RP secondary to SSc who are most likely to struggle with using the app because of problems with hand function. A high proportion (>90%) of participants in both studies had lighter skin tones, meaning that RP (and associated colour changes) in individuals with other skin tones was not well-represented in the data. Further work is urgently needed to determine similarities/differences in RP presentation in those with darker skin tones. Although a high proportion (one-third) of patients did not complete study 1, this may reflect how most were recruited at the outpatient clinic and were perhaps reluctant to attend again only 2 weeks later, and 90% of participants completed study 2. We included only a small number of patients with primary RP because most recent studies of RP have been in patients with SSc-related RP, the form of RP that is the most challenging to treat and therefore most in need of development of new therapies. We recently showed that even patients with SSc whose RP had progressed to digital ulceration were able to take photographs using a smartphone app (not specifically during RP attacks) [28, 29].

In conclusion, our study provides proof of concept for smartphone monitoring of RP attacks, including objective measurement of colour change, and could pave the way for development of an outcome measure (or set of measures) collected by the patient at the time of RP attacks, i.e. when these measures are likely to be most meaningful. The next step is to develop the app further, including easy-to-use options for patients with very impaired hand function, and to validate its use in longitudinal studies including assessment of sensitivity to change in response to vasodilator therapies.

## Supplementary Material

keaf141_Supplementary_Data

## Data Availability

The Sponsor will share de-identified individual participant data collected during the study with researchers who provide a methodologically sound proposal.
