# Supplementary material for: Using a smartphone app to monitor Raynaud’s attacks and quantify skin colour changes—towards objective outcome measures for Raynaud’s
Source: Rheumatology (Oxford). 2025 Mar 12;64(7):4236–44. doi: 10.1093/rheumatology/keaf141 (PMC12212912; doi:10.1093/rheumatology/keaf141)
Supplement: keaf141_Supplementary_Data [file keaf141_supplementary_data.docx]

USING A SMARTPHONE APP TO MONITOR RAYNAUD’S AND QUANTIFY SKIN COLOUR CHANGES – TOWARDS OBJECTIVE OUTCOME MEASURES FOR RAYNAUD’S

Supplementary materials

**Supplementary Data S1. Image analysis**

In order to extract colour information from defined anatomical locations on the surface of the hand it was first necessary to identify the outline of the hand within the digital image. Doing this manually using free-hand drawing software would be extremely time-consuming in a study of any useful size, and so we automated the process of finding the hand outline using a machine learning approach (see Figure 1) using BoneFinder [1], a software tool originally developed for identifying skeletal bone outlines in radiographs. BoneFinder ‘learns’ a deformable model of target shapes (in this case the hand) from a set of training images, using a set of connected points corresponding to anatomical features (finger-tips, joints etc.) to represent the outline (Figure 1(b)). To train the model, we manually marked up the outline of the hand in each of 120 of the collected images. The trained model contained within it the range of natural variation in shape, size and pose of hands, and was subsequently able to find a ‘best fit’ for hand images to which it was naïve. Both left and right hands were accommodated by flipping images around a vertical axis. Using simple geometry we defined 25 regions of interest (ROIs) relative to the hand model points, 24 on the digits and one on the dorsum of the hand, for use as a colour reference (see Figure 1(c)). Once BoneFinder fitted the hand model to a new image, the Red, Green, and Blue (RGB) pixel colour values for each ROI were extracted automatically using a software script written in MATLAB (The Mathworks Inc., Matlab, R2016a).

Colour in digital images can be represented in a number of ways – the standard for digital cameras is RGB, where hue and brightness are intrinsically linked. Since we were interested in colour change during RP episodes rather than changes in brightness (due, for example, to changes in illumination), we transformed (again using a MATLAB script) the pixel RGB values into an alternative CIE L*a*b* colour space [2], where L* is luminance (brightness), while a* and b* are two orthogonal hue axes (red-green and blue-yellow respectively). For each ROI extracted as described previously, we had a large number (several thousand at least) of (usually) similar colour values. Figure 2 shows the distribution of pixel colour values for a typical ROI, plotted in a*b* space at two time points during an RP episode. As the skin in the ROI changes colour the cloud of points shifts. To quantify such shifts or differences between ROIs, we used the Bhattacharyya distance [3, 4], a measure of the similarity between two distributions.

where *q* and *p* are distributions of colour values in colour space (*a*,*b*). To calculate *D*, we used a kernel density estimation method [5, 6] to produce a continuous estimates of *p* and *q* from the point clouds in (*a*,*b*) colour space.

The mean colour change during each RP attack was quantified by the Bhattacharyya distance (abbreviated to BD in main manuscript) in colour space between a region of interest (e.g. a section of a digit) and a control region (dorsal hand) not thought to change during episodes of RP.

**References**

1. Lindner C, Thiagarajah S, Wilkinson JM, The arcOGEN Consortium, Wallis GA, Cootes TF. Fully automatic segmentation of the proximal femur using Random Forest Regression Voting. IEEE Transactions on Medical Imaging 2013; 32: 1462-72.
2. CIE (Commission Internationale de l'Eclairage). Colorimetry - Technical Report. CIE Pub. No. 15 (3^rd^ ed.), Bureau Central de la CIE, Vienna (2004).
3. Bhattacharyya A. On a measure of divergence between two statistical populations defined by their probability distributions. Bull Cal Math Soc 1943; 35:99–110.
4. Bhattacharyya, A. On a measure of divergence between two multinomial populations. Sankhyā: the Indian J Statistics 1946; 7: 401-6.
5. Rosenblatt M. Remarks on some nonparametric estimates of a density function. Ann Math Statist 1956; 27 (3): 832-7.
6. Parzen E. On estimation of a probability density function and mode. Ann Math Statist 1962; 33 (3): 1065–76.

**Supplementary Table S1.** Instructions for photographing RP episodes. The following points were discussed with study participants, as things to consider when photographing their hands.

| Images should be of the whole hand, including all fingers and the thumb. |
| --- |
| During an episode, patients should try to take at least 3 photographs (nominally to cover the start, middle and end of the episode), with ideally one image every 2-3 minutes during the episode.* |
| The same hand (left or right), and the same aspect (dorsal or palmar) should be maintained throughout the episode, although this could be varied between episodes. |
| The X-rite Color Checker card should be included in the photographs wherever practically possible, to allow white balancing and colour correction of the images (Study 1 only). |

*RP attacks naturally vary in duration, both within and between people. For this reason it is difficult to be prescriptive about how many images should be taken to correctly document an attack. The pragmatic approach taken in this work was to allow the patients to determine the best approach based on their symptoms. For the purposes of this work the episodes are defined as starting/ending when the first/last photographs are taken – attack duration can be extracted from comparison of the time stamp data of these images.

**Supplementary Table S2.** Contents and structure of post-attack and daily questionnaires within the Raynaud's monitoring app.

| Raynaud’s App post-attack questionnaire contents and summary of key findings | | |
| --- | --- | --- |
| RP severity | Radio buttons, integer values 0 (minimal symptoms) – 10 (severe symptoms) | Median: 3  Inter-quartile range: 2-4 |
| RP Attack Duration | Longer than normal  Shorter than normal  Usual duration | 105/634 (16.6%)  245/634 (38.6%)  284/634 (44.8%) |
| Trigger | Temperature change  Emotional stress  No trigger | 607/634 (95.7%)  27/634 (4.3%)  0/634 (0%) |
| Did you force the attack to stop? | Yes  No | 164/634 (25.9%)  470/634 (74.1%) |
|  |  |  |
| Raynaud’s App daily questionnaire contents and raw data | | |
| Overall well-being | Slider, integer values 0 (poor) – 10 (great) | Median: 5, inter-quartile range: 3-7 |
| Any changes in your Raynaud’s medication? | Yes/No, answering “Yes” triggers further questions. User can add a new drug, remove a current drug, or change the dose of a current drug. | No changes recorded by patients in the study |
| Do you currently have any finger ulcers? | Yes/No | No patients had ulcers at the start of the study period, or had a new ucler occur during the study. |
| Today you have recorded X attacks with photos, how many others have you had without taking photos? | User enters number of attacks, integer value must be 0 or greater. | This option was used 17 times during the study, by 5 different patients. Maximum number of non-photographed attacks in a single day was 2. |
| Please take a photo of your hand | User takes picture of hand at time of questionnaire completion. | Few patients used this option, and the images collected were not further utilised |


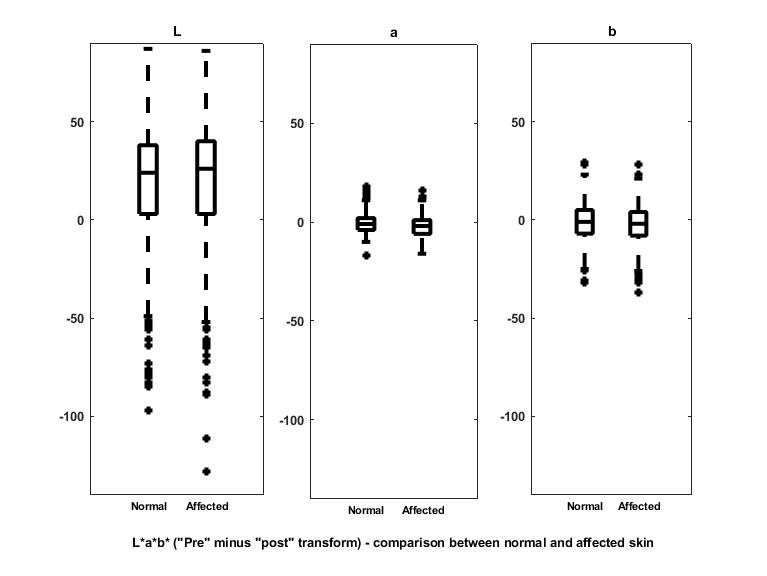


**Supplementary Figure S1**. Assessing the colour checker card. Change due to colour correction in the value of the three colour channel components (L*, a*, and b*) of normal skin and skin affected during a Raynaud's attack. Each panel shows box and whisker plots for normal and RP-affected skin. The data being represented is the difference between raw and colour-corrected pixel values. Since the average values approximately centre round zero there is no difference pre and post correction and therefore the use of the colour checker card is not required for these images.
